# Experimental data on radio frequency interference in microwave links using frequency scan measurements at 6 GHz, 7 GHz, and 8 GHz

**DOI:** 10.1016/j.dib.2021.106916

**Published:** 2021-02-26

**Authors:** Glory Uzuazobona Ughegbe, Michael Adedosu Adelabu, Agbotiname Lucky Imoize

**Affiliations:** a Department of Electrical and Electronics Engineering, Faculty of Engineering, University of Lagos, Akoka 100213, Lagos, Nigeria; b Department of Electrical Engineering and Information Technology, Institute of Digital Communication, Ruhr University, 44801 Bochum, Germany

**Keywords:** Radio frequency interference, Frequency availability, Frequency scanning, Horizontal and vertical polarizations, Frequency band, Frequency reuse, Frequency range

## Abstract

One of the biggest challenges for wireless communication network operators is how to minimize or mitigate radio frequency interference (RFI) for efficient network services at the desired quality of service (QoS). Microwave radio links are highly susceptible to interference from narrow and wideband sources. Interference ultimately affects network quality and contributes to the colossal loss of usable mobile data, leading to substantial operational costs for network operators. Additionally, the implementation of high capacity microwave links could potentially force the channels to point towards the same direction, posing a significant interference source. Radio frequency interference issues on the microwave links should be prioritized for prompt resolution or mitigation to achieve the minimum QoS requirement for the growing network subscribers. Toward this end, frequency scans are required to accurately picture the available frequency plan and channels based on the allocated spectrum. This article presents experimental data on radio frequency interference of active microwave links at 6 GHz, 7 GHz, and 8 GHz. The extensive frequency scans were obtained from eighteen active base stations located in Kogi, Lagos, and Rivers States in Nigeria. The frequency scans were carried out using the Anritsu MS2724C spectrum analyzer and a 0.6-meter antenna dish with full azimuth coverage. The analyzer captures the horizontal and vertical polarization. The frequency scan measurements reported in this article would be significantly useful to radio frequency interference detection and mitigation, preliminary network equipment positioning, frequency selection and assignment, and microwave network planning.

## Specifications Table

**Table:** 

Subject	Engineering and Technology
Specific subject area	Wireless Communication; Microwave Engineering
Type of data	Graph, Tables
How data were acquired	The measured data presented in this data article were acquired through extensive frequency scans on eighteen base stations (BS) located in Kogi, Lagos, and Rivers States of Nigeria. The frequency scans were carried out using Anritsu MS2724C spectrum analyzer and a 0.6-meter antenna dish with full azimuth coverage at 6 GHz, 7 GHz, and 8 GHz. The measured data was automatically saved on the spectrum analyzer at every one hour for further processing.
Data format	Raw and Analyzed
Parameters for data collection	The critical parameters measured include; tower azimuth showing the vertical and horizontal polarizations, frequency-domain feature depicted on the x-axis as the frequency, and the y-axis as the power or amplitude domain of the subcarrier in dBm. The output of the spectrum analyzer is an X-Y trace on display. The display is mapped on a grid (graticule) with ten major horizontal divisions and generally ten major vertical divisions. The horizontal axis is linearly calibrated in frequency that increases from left to right, whereas the vertical axis is calibrated in amplitude. Setting the frequency was carried out in a two-step process. First, the frequency at the centerline of the graticule with the center frequency control was adjusted. Next, the frequency range (span) across the full ten divisions with the frequency span control was adjusted. These controls are independent, implying that changing the center frequency does not significantly alter the frequency span. Setting the start and stop frequencies instead of the center frequency and span was considered in some scenarios. In either case, the absolute frequency of any signal displayed and the relative frequency difference between any two signals was evaluated.
Description of data collection	The experimental data were collected at 6 GHz, 7 GHz, and 8 GHz from the tested microwave links using the spectrum analyzer, which was allowed to sweep for about 24 h. The measurements were saved automatically to the spectrum analyzer at every one hour. The readings are extracted to a computer as raw data for further analysis, and this procedure was repeated for all the selected sites of the tested radio links.
Data source location	The data reported in this article were collected from eighteen (18) BSs with the following nomenclature and coordinates; Site A (Latitude 07 55 05.6 N; Longitude 006 16 05.8E), Site B (Latitude 07 59 35.1 N; Longitude 006 37 56.4E), Site C (Latitude 08 04 28.6 N; Longitude 005 45 58.1E), Site D (Latitude 07 54 16.2 N; Longitude 006 02 14.2E), Site E (Latitude 08 13 04.3 N; Longitude 005 30 27.6E), Site F (Latitude 08 04 28.6 N; Longitude 005 45 58.1E), Site G (Latitude 07 59 35.1 N; Longitude 006 37 56.4 E), Site H (Latitude 07 49 24.6 N; Longitude 006 44 07.4E), Site I (Latitude 08 10 03.7 N; Longitude 005 04 08.0E), Site J (Latitude 08 09 13.0 N; Longitude 004 42 35.6E), Site K (Latitude 08 13 04.3 N; Longitude 005 30 27.6E), Site L (Latitude 08 11 25.8 N; Longitude 005 14 38.4E), Site M (Latitude 08 10 03.7 N; Longitude 005 04 08.0E), Site N (Latitude 08 11 25.8 N; Longitude 005 14 38.4E), Site P (Latitude 06 28 58.0 N; Longitude 003 47 31.3E), Site Q (Latitude 06 29 10.4 N; Longitude 003 52 43.7E), Site R (Latitude 06 34 58.9 N; Longitude 003 58 31.2E), and Site S (Latitude 06 28 58.0 N; Longitude 003 47 31.3E).
Data accessibility	Detailed measured data on the frequency scans is provided as a supplementary file attached to this data article in a spreadsheet format for easy accessibility and reusability.

## Value of the Data

• The data reported in this article will provide valuable information to enhance the performance of microwave link propagation in related environments.
• The data presented will be of immense benefit to researchers in the field of microwave propagation, especially for radio frequency interference (RFI) analysis.
• The data reported in this article will benefit radio frequency (RF) or field engineers during RFI troubleshooting and fault resolution in microwave links.
• The data will help in key performance indicators (KPI) analysis, accurate frequency planning and assignment during radio link deployment and implementation.
• The frequency scan reports will support further insights towards the development of experimental procedures to accelerate research in Microwave Engineering.

## 1 Data Description

Radio frequency interference (RFI) [1] is a challenging problem in microwave engineering, and particularly in radio waves propagation, with so many uncertainties surrounding the ability to deal with the enormous threats it presents. Specifically, RFI poses a considerable problem to network operators, and its attendant revenue depletion fuels the need for detailed frequency scans on all microwave links deployed in the network [2]. The uncertainty and vulnerability of wireless network services to RFI from mainly unlicensed radio bands are alarming. RFI triggers churn of such mobile services by customers for reliable and efficient network solutions. Potential RFI sources include but are not limited to spurious signals from lower frequency bands and noise from different coherent or incoherent interference waves [3,4].

The potential solution to the RFI problem is to ensure proper frequency scanning during deployment to monitor the presence of RFI and quickly localize it upon detection. Apart from this, frequency scanning is used during corrective maintenance for RFI related issues and wireless network failure [5,6].

The implementation of high capacity microwave links could potentially force the channels to point towards the same direction, posing a potential source of interference [7,8]. Detection and mitigation of radio frequency interference in microwave links – frequency scans before microwave link deployment help operators detect existing interference and other RF jamming sources. The frequency scans reported in this article would support the analysis of the assigned frequency spectrum and channels and determine the given frequency availability. The frequency scans would provide a clear picture of the available frequency plan and channels based on the allocated spectrum. Also, the frequency scans would play a key role in aiding the rapid deployment of wireless network services [9,10].

**Fig. 1:**
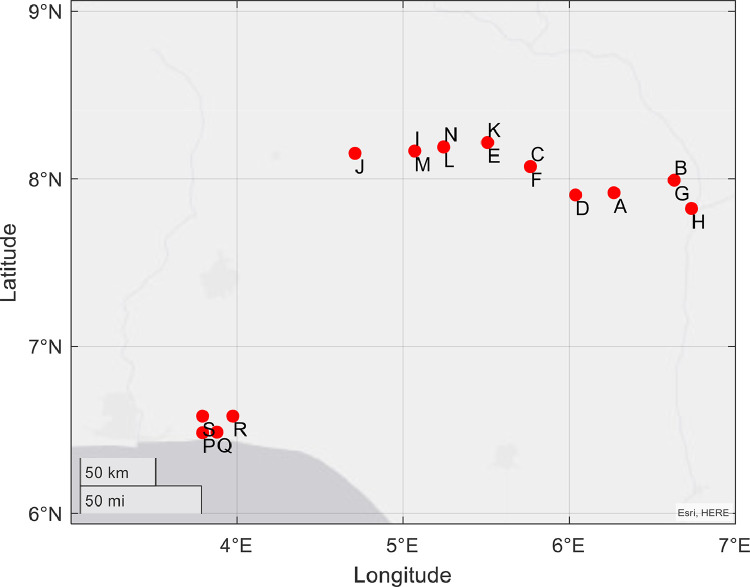
This is a graph showing the geographical coordinates of the environment where frequency scan measurements were taken.

This data article focuses on the importance of frequency scanning as a deployment, preventive, and corrective tool for optimum microwave radio link performance for the benefits of the network operators and subscribers. Toward this end, extensive frequency scans were carried out in three States of Nigeria; Kogi, Lagos, and Rivers. The States lie in three different geographical zones representing South-West, North-Central, and South-South of Nigeria. A graph showing the geographical coordinates of the environment where frequency scan measurements were taken is given in Fig. 1.

The frequency scans were taken in and around the selected eighteen base stations at 6 GHz, 7 GHz, and 8 GHz bands, considering the horizontal and vertical polarizations, using a 0.6-meter antenna and a spectrum analyzer [11]. Finally, the measured parameters were saved on the analyzer and extracted to a personal computer for processing, and data analysis was carried out in MATLAB.

The parameters derived from the experimental data are briefly described as follows. The frequency usage plan reports for site A until site S are presented in Table 1, Table 2, Table 3, Table 4, Table 5, Table 6, Table 7, Table 8, Table 9. These cover the eighteen base stations investigated. In particular, Table 1 covers site A and site B (radio link 1), and all the channels are available for the 6 GHz and 8 GHz bands for use by licensed mobile operators. Table 2 presents site C and site D (radio link 2), and Table 3 reports site E and site F (radio link 3). Specifically, high noise is observed on the 6 GHz band, and all 8 GHz channels are available for these links. However, only channel 3 is available on the 7 GHz band for the horizontal and vertical polarization. As observed in Table 4, site G and site H (radio link 4) show that only channel 2 is available on the 6 GHz band while noise exists on its other channels. Interestingly, three channels and two channels are available for the 7 GHz and 8 GHz bands, respectively, for the tested radio link 4.

**Table 1:** Frequency usage plan report for site A and site B (Radio Link 1).

Frequency (6 GHz)	CH. Nos	Center Frequency (MHz)	Result	V	H	CH. RX
**SITE A**						

Start - 5900 MHz	1	5945.200	Available	A	A	1L
End - 6500 MHz	2	5974.850	Available	A	A	2L
	3	6004.500	Available	A	A	3L
	4	6034.150	Available	A	A	4L
	5	6093.450	Available	A	A	5L
	6	6152.750	Available	A	A	6L
Frequency (7 GHz)	1	7457.000	Not Available	NA	NA	1L
Start - 7400 MHz	2	7485.000	Available	A	A	2L
End - 7800 MHz	3	7513.000	Available	A	A	3L
	4	7541.000	Available	A	A	4L
	5	7569.000	Available	A	A	5L
Frequency (8 GHz)	4	7836.650	Available	A	A	4L
Start - 7700 MHz	7	7925.600	Available	A	A	7L
End - 8300 MHz	8	7955.250	Available	A	A	8L

**SITE B**						

Start - 5900 MHz	1	6197.240	Available	A	A	1H
End - 6500 MHz	2	6226.890	Available	A	A	2H
	3	6256.540	Available	A	A	3H
	4	6286.190	Available	A	A	4H
	5	6345.490	Available	A	A	5H
	6	6375.140	Available	A	A	6H
Frequency (7 GHz)	1	7625.000	Not Available	NA	A	1H
Start - 7400 MHz	2	7653.000	Not Available	NA	A	2H
End - 7800 MHz	3	7681.000	Available	A	A	3H
	4	7709.000	Available	A	A	4H
	5	7737.000	Available	A	A	5H
Frequency (8 GHz)	4	8147.970	Available	A	A	4H
Start – 7700MHz	7	8236.920	Available	A	A	7H
End - 8300 MHz	8	8266.570	Available	A	A	8H

**Note** – Available: A; Not Available: NA; Vertical: V, Horizontal: H.

**Table 2:** Frequency usage plan report for site C and site D (Radio Link 2).

Frequency (6 GHz)	CH. Nos	Center Frequency (MHz)	Result	V	H	CH. RX
**SITE C**						

Start - 5900 MHz	1	5945.200	Not Available	NA	NA	1L
End - 6500 MHz	2	5974.850	Not Available	NA	NA	2L
	3	6004.500	Not Available	NA	NA	3L
	4	6034.150	Not Available	NA	NA	4L
	5	6093.450	Not Available	NA	NA	5L
	6	6152.750	Not Available	NA	NA	6L
Frequency (7 GHz)	1	7457.000	Not Available	A	NA	1L
Start - 7400 MHz	2	7485.000	Not Available	A	NA	2L
End - 7800 MHz	3	7513.000	Available	A	A	3L
	4	7541.000	Available	A	A	4L
	5	7569.000	Available	A	A	5L
Frequency (8 GHz)	4	7836.650	Available	A	A	4L
Start - 7700 MHz	7	7925.600	Available	A	A	7L
End - 8300 MHz	8	7955.250	Available	A	A	8L

**SITE D**						

Start - 5900 MHz	1	6197.240	Not Available	NA	NA	1H
End - 6500 MHz	2	6226.890	Not Available	NA	NA	2H
	3	6256.540	Not Available	NA	NA	3H
	4	6286.190	Not Available	NA	NA	4H
	5	6345.490	Not Available	NA	NA	5H
	6	6375.140	Not Available	NA	NA	6H
Frequency (7 GHz)	1	7625.000	Not Available	NA	NA	1H
Start - 7400 MHz	2	7653.000	Not Available	A	NA	2H
End - 7800 MHz	3	7681.000	Available	A	A	3H
	4	7709.000	Not Available	NA	NA	4H
	5	7737.000	Not Available	NA	A	5H
					
Frequency (8 GHz)	4	8147.970	Available	A	A	4H
Start - 7700 MHz	7	8236.920	Available	A	A	7H
End - 8300 MHz	8	8266.570	Available	A	A	8H

**Note** – Available: A; Not Available: NA; Vertical: V, Horizontal: H.

**Table 3:** Frequency usage plan report for site E and site F (Radio Link 3).

Frequency (6 GHz)	CH. Nos	Center Frequency (MHz)	Result	V	H	CH. RX
**SITE E**						

Start - 5900 MHz	1	5945.200	Not Available	NA	NA	1L
End - 6500 MHz	2	5974.850	Not Available	NA	NA	2L
	3	6004.500	Not Available	NA	NA	3L
	4	6034.150	Not Available	NA	NA	4L
	5	6093.450	Not Available	NA	NA	5L
	6	6152.750	Not Available	NA	NA	6L
Frequency (7 GHz)	1	7457.000	Not Available	A	NA	1L
Start - 7400 MHz	2	7485.000	Not Available	A	NA	2L
End - 7800 MHz	3	7513.000	Available	A	A	3L
	4	7541.000	Available	A	A	4L
	5	7569.000	Available	A	A	5L
Frequency (8 GHz)	4	7836.650	Available	A	A	4L
Start - 7700 MHz	7	7925.600	Available	A	A	7L
End - 8300 MHz	8	7955.250	Available	A	A	8L

**SITE F**						

Start - 5900 MHz	1	6197.240	Not Available	NA	NA	1H
End - 6500 MHz	2	6226.890	Not Available	NA	NA	2H
	3	6256.540	Not Available	NA	NA	3H
	4	6286.190	Not Available	NA	NA	4H
	5	6345.490	Not Available	NA	NA	5H
	6	6375.140	Not Available	NA	NA	6H
Frequency (7 GHz)	1	7625.000	Not Available	NA	NA	1H
Start - 7400 MHz	2	7653.000	Not Available	NA	NA	2H
End - 7800 MHz	3	7681.000	Available	A	A	3H
	4	7709.000	Not Available	NA	NA	4H
	5	7737.000	Not Available	NA	NA	5H
Frequency (8 GHz)	4	8147.970	Available	A	A	4H
Start - 7700 MHz	7	8236.920	Available	A	A	7H
End - 8300 MHz	8	8266.570	Available	A	A	8H

**Note** – Available: A; Not Available: NA; Vertical: V, Horizontal: H.

**Table 4:** Frequency usage plan report for site G and site H (Radio Link 4).

Frequency (6 GHz)	CH. Nos	Center Frequency (MHz)	Result	V	H	CH. RX
**SITE G**						

Start - 5900 MHz	1	5945.200	Available	A	A	1L
End - 6500 MHz	2	5974.850	Available	A	A	2L
	3	6004.500	Available	A	A	3L
	4	6034.150	Available	A	A	4L
	5	6093.450	Available	A	A	5L
	6	6152.750	Available	A	A	6L
						
Frequency (7 GHz)	1	7457.000	Not Available	NA	NA	1L
Start - 7400 MHz	2	7485.000	Available	A	A	2L
End - 7800 MHz	3	7513.000	Not Available	NA	NA	3L
	4	7541.000	Available	A	A	4L
	5	7569.000	Available	A	A	5L
						
Frequency (8 GHz)	4	7836.650	Not Available	A	NA	4L
Start - 7700 MHz	7	7925.600	Available	A	A	7L
End - 8300 MHz	8	7955.250	Available	A	A	8L

**SITE H**						

Start - 5900 MHz	1	6197.240	Not Available	NA	NA	1H
End - 6500 MHz	2	6226.890	Available	A	A	2H
	3	6256.540	Not Available	NA	NA	3H
	4	6286.190	Not Available	A	NA	4H
	5	6345.490	Not Available	NA	NA	5H
	6	6375.140	Not Available	NA	NA	6H
Frequency (7 GHz)	1	7625.000	Not Available	A	NA	1H
Start - 7400 MHz	2	7653.000	Available	A	A	2H
End - 7800 MHz	3	7681.000	Not Available	A	NA	3H
	4	7709.000	Available	A	A	4H
	5	7737.000	Available	A	A	5H
Frequency (8 GHz)	4	8147.970	Available	A	A	4H
Start - 7700 MHz	7	8236.920	Available	A	A	7H
End - 8300 MHz	8	8266.570	Available	A	A	8H

**Note** – Available: A; Not Available: NA; Vertical: V, Horizontal: H.

**Table 5:** Frequency usage plan report for site I and site J (Radio Link 5).

Frequency (6 GHz)	CH. Nos	Center Frequency (MHz)	Result	V	H	CH. RX
**SITE I**						

Start - 5900 MHz	1	5945.200	Not Available	NA	NA	1L
End - 6500 MHz	2	5974.850	Not Available	NA	NA	2L
	3	6004.500	Not Available	NA	NA	3L
	4	6034.150	Not Available	NA	NA	4L
	5	6093.450	Not Available	NA	NA	5L
	6	6152.750	Not Available	NA	NA	6L
Frequency (7 GHz)	1	7457.000	Not Available	NA	NA	1L
Start - 7400 MHz	2	7485.000	Not Available	NA	A	2L
End - 7800 MHz	3	7513.000	Not Available	A	NA	3L
	4	7541.000	Available	A	A	4L
	5	7569.000	Not Available	NA	NA	5L
Frequency (8 GHz)	4	7836.650	Not Available	A	NA	4L
Start - 7700 MHz	7	7925.600	Not Available	NA	A	7L
End - 8300 MHz	8	7955.250	Not Available	A	NA	8L

**SITE J**						

Start - 5900 MHz	1	6197.240	Not Available	A	NA	1H
End - 6500 MHz	2	6226.890	Available	A	A	2H
	3	6256.540	Available	A	A	3H
	4	6286.190	Available	A	A	4H
	5	6345.490	Available	A	A	5H
	6	6375.140	Not Available	A	NA	6H
Frequency (7 GHz)	1	7625.000	Not Available	NA	NA	1H
Start - 7400 MHz	2	7653.000	Not Available	NA	NA	2H
End - 7800 MHz	3	7681.000	Not Available	NA	NA	3H
	4	7709.000	Available	A	A	4H
	5	7737.000	Not Available	NA	A	5H
Frequency (8 GHz)	4	8147.970	Available	A	A	4H
Start - 7700 MHz	7	8236.920	Available	A	A	7H
End - 8300 MHz	8	8266.570	Available	A	A	8H

**Note** – Available: A; Not Available: NA; Vertical: V, Horizontal: H.

**Table 6:** Frequency usage plan report for site K and site L (Radio Link 6).

Frequency (6 GHz)	CH. Nos	Center Frequency (MHz)	Result	V	H	CH. RX
**SITE K**						

Start - 5900 MHz	1	5945.200	Not Available	NA	NA	1L
End - 6500 MHz	2	5974.850	Available	A	A	2L
	3	6004.500	Not Available	NA	A	3L
	4	6034.150	Available	A	A	4L
	5	6093.450	Available	A	A	5L
	6	6152.750	Available	A	A	6L
Frequency (7 GHz)	1	7457.000	Not Available	A	NA	1L
Start - 7400 MHz	2	7485.000	Not Available	A	NA	2L
End - 7800 MHz	3	7513.000	Available	A	A	3L
	4	7541.000	Not Available	A	NA	4L
	5	7569.000	Not Available	A	NA	5L
Frequency (8 GHz)	4	7836.650	Available	A	A	4L
Start - 7700 MHz	7	7925.600	Not Available	A	NA	7L
End - 8300 MHz	8	7955.250	Available	A	A	8L

**SITE L**						

Start - 5900 MHz	1	6197.240	Available	A	A	1H
End - 6500 MHz	2	6226.890	Available	A	A	2H
	3	6256.540	Available	A	A	3H
	4	6286.190	Available	A	A	4H
	5	6345.490	Available	A	A	5H
	6	6375.140	Available	A	A	6H
Frequency (7 GHz)	1	7625.000	Not Available	A	NA	1H
Start - 7400 MHz	2	7653.000	Not Available	A	NA	2H
End - 7800 MHz	3	7681.000	Available	A	A	3H
	4	7709.000	Not Available	A	NA	4H
	5	7737.000	Not Available	A	A	5H
Frequency (8 GHz)	4	8147.970	Available	A	A	4H
Start - 7700 MHz	7	8236.920	Available	A	A	7H
End - 8300 MHz	8	8266.570	Available	A	A	8H

**Note** – Available: A; Not Available: NA; Vertical: V, Horizontal: H.

**Table 7:** Frequency usage plan report for site M and site N (Radio Link 7).

Frequency (6 GHz)	CH. Nos	Center Frequency (MHz)	Result	V	H	CH. RX
**SITE M**						

Start - 5900 MHz	1	5945.200	Not Available	NA	NA	1L
End - 6500 MHz	2	5974.850	Not Available	NA	NA	2L
	3	6004.500	Not Available	NA	NA	3L
	4	6034.150	Not Available	NA	NA	4L
	5	6093.450	Not Available	NA	NA	5L
	6	6152.750	Not Available	NA	NA	6L
Frequency (7 GHz)	1	7457.000	Not Available	NA	NA	1L
Start - 7400 MHz	2	7485.000	Not Available	NA	NA	2L
End - 7800 MHz	3	7513.000	Available	A	A	3L
	4	7541.000	Not Available	NA	NA	4L
	5	7569.000	Available	A	A	5L
Frequency (8 GHz)	4	7836.650	Not Available	NA	NA	4L
Start - 7700 MHz	7	7925.600	Available	A	A	7L
End - 8300 MHz	8	7955.250	Available	A	A	8L

**SITE N**						

Start - 5900 MHz	1	6197.240	Available	A	A	1H
End - 6500 MHz	2	6226.890	Available	A	A	2H
	3	6256.540	Available	A	A	3H
	4	6286.190	Available	A	A	4H
	5	6345.490	Available	A	A	5H
	6	6375.140	Available	A	NA	6H
Frequency (7 GHz)	1	7625.000	Not Available	NA	A	1H
Start - 7400 MHz	2	7653.000	Not Available	NA	NA	2H
End - 7800 MHz	3	7681.000	Available	A	A	3H
	4	7709.000	Not Available	NA	NA	4H
	5	7737.000	Not Available	A	A	5H
Frequency (8 GHz)	4	8147.970	Available	A	A	4H
Start - 7700 MHz	7	8236.920	Available	A	A	7H
End - 8300 MHz	8	8266.570	Available	A	A	8H

**Note** – Available: A; Not Available: NA; Vertical: V, Horizontal: H.

**Table 8:** Frequency usage plan report for site P and site Q (Radio Link 8).

Frequency (6 GHz)	CH. Nos	Center Frequency (MHz)	Result	V	H	CH. RX
**SITE P**						

Start - 5900 MHz	1	5945.200	Not Available	NA	NA	1L
End - 6500 MHz	2	5974.850	Available	A	A	2L
	3	6004.500	Not Available	NA	NA	3L
	4	6034.150	Available	A	A	4L
	5	6093.450	Available	A	A	5L
	6	6152.750	Available	A	A	6L
Frequency (7 GHz)	1	7457.000	Not Available	NA	NA	1L
Start - 7400 MHz	2	7485.000	Not Available	NA	NA	2L
End - 7700 MHz	3	7513.000	Not Available	NA	NA	3L
	4	7541.000	Not Available	NA	NA	4L
	5	7569.000	Not Available	NA	NA	5L
Frequency (8 GHz)	4	7836.650	Not Available	NA	NA	4L
Start - 7800 MHz	7	7925.600	Not Available	NA	NA	7L
End - 8300 MHz	8	7955.250	Not Available	NA	NA	8L

**SITE Q**						

Start - 5900 MHz	1	6197.240	Available	A	A	1H
End - 6500 MHz	2	6226.890	Not Available	A	NA	2H
	3	6256.540	Available	A	A	3H
	4	6286.190	Available	A	A	4H
	5	6345.490	Not Available	A	NA	5H
	6	6375.140	Not Available	NA	NA	6H
Frequency (7 GHz)	1	7625.000	Not Available	NA	NA	1H
Start - 7400 MHz	2	7653.000	Available	A	A	2H
End - 7700 MHz	3	7681.000	Not Available	NA	NA	3H
	4	7709.000	Not Available	NA	NA	4H
	5	7737.000	Not Available	NA	NA	5H
Frequency (8 GHz)	4	8147.970	Available	NA	NA	4H
Start - 7800 MHz	7	8236.920	Available	A	A	7H
End - 8300 MHz	8	8266.570	Available	NA	NA	8H

**Note** – Available: A; Not Available: NA; Vertical: V, Horizontal: H.

**Table 9:** Frequency usage plan report for site R and site S (Radio Link 9).

Frequency (6 GHz)	CH. Nos	Center Frequency (MHz)	Result	V	H	CH. RX
**SITE R**						

Start - 5900 MHz	1	5945.200	Available	A	A	1L
End - 6500 MHz	2	5974.850	Available	A	A	2L
	3	6004.500	Available	A	A	3L
	4	6034.150	Available	A	A	4L
	5	6093.450	Available	A	A	5L
	6	6152.750	Available	A	A	6L
Frequency (7 GHz)	1	7457.000	Not Available	NA	NA	1L
Start - 7400 MHz	2	7485.000	Available	A	A	2L
End - 7700 MHz	3	7513.000	Not Available	NA	NA	3L
	4	7541.000	Not Available	A	NA	4L
	5	7569.000	Not Available	NA	NA	5L
Frequency (8 GHz)	4	7836.650	Not Available	NA	NA	4L
Start - 7800 MHz	7	7925.600	Not Available	NA	NA	7L
End - 8300 MHz	8	7955.250	Not Available	NA	NA	8L

**SITE S**						

Start - 5900 MHz	1	6197.240	Not Available	NA	NA	1H
End - 6500 MHz	2	6226.890	Available	A	A	2H
	3	6256.540	Available	A	A	3H
	4	6286.190	Available	A	A	4H
	5	6345.490	Available	A	A	5H
	6	6375.140	Not Available	NA	NA	6H
Frequency (7 GHz)	1	7625.000	Not Available	NA	NA	1H
Start - 7400 MHz	2	7653.000	Available	A	A	2H
End - 7700 MHz	3	7681.000	Not Available	NA	NA	3H
	4	7709.000	Available	A	A	4H
	5	7737.000	Not Available	NA	NA	5H
Frequency (8 GHz)	4	8147.970	Not Available	NA	NA	4H
Start - 7800 MHz	7	8236.920	Not Available	NA	A	7H
End - 8300 MHz	8	8266.570	Not Available	NA	NA	8H

**Note** – Available: A; Not Available: NA; Vertical: V, Horizontal: H.

The frequency scans for radio link 5 comprising site I and site J are presented in Table 5. High noise exists on all the frequency bands, and this link appears not to be useful. Table 6 shows site K and site L (radio link 6). The channels are more available on the vertical polarization than on the horizontal polarization for the tested 6 GHz, 7 GHz, and 8 GHz frequency bands. For site M and site N (radio link 7) reported in Table 7, only three channels are available on the investigated frequency bands. Table 8 presents the frequency scan for site P and site Q (radio link 8), and most channels are available on all the frequency bands, except channel eight and channel five on the 6 GHz and 7 GHz, respectively. Moreover, high noise is observed on the 8 GHz band of site R and site S (radio link 9), as reported in Table 9.

## 2 Experimental Design, Materials and Methods

The equipment used for the measurements campaign is the Anritsu Spectrum Master MS2724C and a 0.6-meter antenna dish. The measurements were taken at 6 GHz, 7 GHz, and 8 GHz with full azimuth coverage. In particular, the spectrum analyzer was used to test and troubleshoot radio frequency-related issues in the investigated environment. It was also used for detecting spurious signals and measuring signal bandwidth by displaying amplitude against frequency.

During the measurements campaign, two selections were made to set the frequency of the spectrum analyzer. These are the ‘‘start and stop’’ or ‘‘top and bottom’’ frequencies. These frequencies were used for the sweep exercise or test. The spectrum analyzer scans the required frequency span from the low to the high end of the range. In this case, the speed of the scan is an essential factor. Precisely, the faster it scans the range, the quicker the measurement duration is completed. Further to this, the spectrum scanning mode feature was used to analyze the signal amplitude (strength) as it varies by signal frequency in the uplink. In simple terms, a scanned graph represents what is happening in the RF spectrum and helps in determining the best frequency to use and where there may be severe interference.

The frequency scan measurements comprise the In-phase and Quadrature (I/Q) data. The I/Q data uses time/amplitude and frequency information processed by the baseband processing unit of the measurement equipment. Here, a time or amplitude domain function is described in terms of an associated frequency spectrum. The scanning process consists of convolving, or “sliding,” a spectrum “window” across the frequency spectrum as an amplitude function.

Finally, the spectrum master was connected to the antenna fixed on a specific location and allowed to sweep for twenty-four (24) hours. The reading is then automatically saved on the Anritsu spectrum analyzer every one hour. The measured data was extracted to a Lenovo T430 personal computer for further processing, and the analysis was done in MATLAB, a product of Mathworks Incorporated.

## Ethics Statement

The work did not involve the use of human subjects nor animal experiments.

## CRediT Author Statement

**Glory Uzuazobona Ughegbe** and **Agbotiname Lucky Imoize:** Conceptualization, Methodology, Software; **Agbotiname Lucky Imoize:** Data curation, Writing – Original draft preparation; **Glory Uzuazobona Ughegbe, Michael Adedosu Adelabu** and **Agbotiname Lucky Imoize:** Investigation; **Michael Adedosu Adelabu:** Supervision; **Agbotiname Lucky Imoize** and **Michael Adedosu Adelabu:** Writing-Reviewing and Editing.

## Declaration of Competing Interest

The authors declare that they have no known competing financial interests or personal relationships, which have, or could be perceived to have, influenced the work reported in this article.

## Appendix A: Supplementary Material

Supplementary data related to this article can be seen as an attached file for research reproduction and data reusability.

## Appendix C Supplementary materials

Image, application 1
